# Effectiveness of non-pharmaceutical interventions to contain COVID-19: a case study of the 2020 spring pandemic wave in New York City

**DOI:** 10.1098/rsif.2020.0822

**Published:** 2021-02-23

**Authors:** Wan Yang, Jaimie Shaff, Jeffrey Shaman

**Affiliations:** ^1^ Department of Epidemiology, Mailman School of Public Health, Columbia University, New York, NY 10032, USA; ^2^ New York City Department of Health and Mental Hygiene, New York, NY, USA; ^3^ Department of Environmental Health Sciences, Mailman School of Public Health, Columbia University, New York, NY, USA

**Keywords:** COVID-19, transmission dynamics, effectiveness of intervention, social distancing, lockdown, face covering

## Abstract

As COVID-19 continues to pose significant public health threats, quantifying the effectiveness of different public health interventions is crucial to inform intervention strategies. Using detailed epidemiological and mobility data available for New York City and comprehensive modelling accounting for under-detection, we reconstruct the COVID-19 transmission dynamics therein during the 2020 spring pandemic wave and estimate the effectiveness of two major non-pharmaceutical interventions—lockdown-like measures that reduce contact rates and universal masking. Lockdown-like measures were associated with greater than 50% transmission reduction for all age groups. Universal masking was associated with an approximately 7% transmission reduction overall and up to 20% reduction for 65+ year olds during the first month of implementation. This result suggests that face covering can substantially reduce transmission when lockdown-like measures are lifted but by itself may be insufficient to control SARS-CoV-2 transmission. Overall, findings support the need to implement multiple interventions simultaneously to effectively mitigate COVID-19 spread before the majority of population can be protected through mass-vaccination.

## 1. Introduction

Since the emergence of SARS-CoV-2 in late 2019, the virus has infected over 79 million people and killed over 1.75 million worldwide by the end of 2020 (as of 27 December 2020) [1]. Non-pharmaceutical interventions such as social distancing and face covering have been the main strategies to contain COVID-19 during this pandemic in 2020. In late 2020, Phase-III trials for several SARS-CoV-2 vaccines showed highly promising results and were granted emergency use in several countries [2,3]. However, before these vaccines become widely available to the general population (likely in mid- or late 2021), non-pharmaceutical interventions will need to remain the main strategies to contain COVID-19. In addition, future (re)emerging infectious disease outbreaks may need to rely on similar non-pharmaceutical measures. It is thus critical to understand the effectiveness of different non-pharmaceutical interventions implemented during the COVID-19 pandemic waves in order to inform effective future planning while balancing economic need. For instance, with face covering and social distancing by closing businesses as two main interventions, the more effective face covering is, the more businesses could remain open. As a simplified calculation, with a basic reproductive number (*R_*0*_*) of 3 and minimal immunity, a city could maintain 55% business capacity while curbing epidemic growth, if its residents could reduce transmission by 40% using face covering (i.e. effective reproductive number *R_t_* = 3 × 55% × (1–40%) = 0.99 < 1); this threshold business capacity would drop to 33% if no residents used face covering.

However, assessing the effectiveness and impact of a given intervention for COVID-19 has been challenging due to low infection detection rates (many asymptomatic and mild infections do not seek care or receive testing) [4], fluctuation of those infection detection rates, differential disease manifestation by age group [5,6] and concurrent public health interventions. As such, while a few studies have assessed the overall effectiveness of lockdown-like measures, to date, the effectiveness of specific measures including face covering *under real-world conditions* remains unclear.

To estimate the effectiveness of different non-pharmaceutical public health interventions, here we thus focus on the 2020 spring COVID-19 pandemic wave in New York City (NYC), the first COVID-19 epidemic centre in the USA, where detailed data are also available. NYC experienced widespread COVID-19 transmission citywide since early March and recorded over 200 000 cases and over 21 000 COVID-19 confirmed or probable deaths during the following three months. To curb this intense transmission, NY State and NYC implemented multiple intervention measures, including health promotion campaigns in early March, telecommuting and staggered work schedule recommendations beginning the week of 8 March public schools closure starting the week of 15 March [7], stay-at-home orders for non-essential workers starting the week of 22 March [8], and requirements for use of face covering in public starting the week of 12 April [9]. With these overlapping and far-reaching public health interventions, case diagnoses and hospitalizations peaked in April and started to decline substantially in late April and May. NYC was able to begin its phased re-opening of industries starting the week of 7 June 2020.

In this study, we apply a model-inference system [10–12] developed to support the city's COVID-19 pandemic response to reconstruct the underlying transmission dynamics of COVID-19 in NYC during 1 March–6 June 2020 (i.e. prior to the city's reopening). To address the aforementioned challenges, our model-inference system simultaneously assimilates three sources of data: (i) confirmed COVID-19 case data, (ii) COVID-19 associated death data (both cases and deaths are assimilated by neighbourhood and age group) and (iii) neighbourhood-level mobility data to constrain the model system. This enables inference of the overall infection rate (i.e. including those not documented by surveillance), estimation of key transmission characteristics (e.g. the reproductive number) through time and assessment of the effectiveness of different public health interventions, including social distancing and face covering, implemented over time. We further incorporate these estimates to project cases and deaths in the weeks beyond our study period and compare the projections to independent observations in order to evaluate the accuracy of these estimates. We conclude with a discussion on the implication of our findings on strategies to safely reopen economies in places COVID-19 continues to pose substantial public health threats.

## 2. Results

### 2.1. Overall epidemic trends

**Figure 1.**
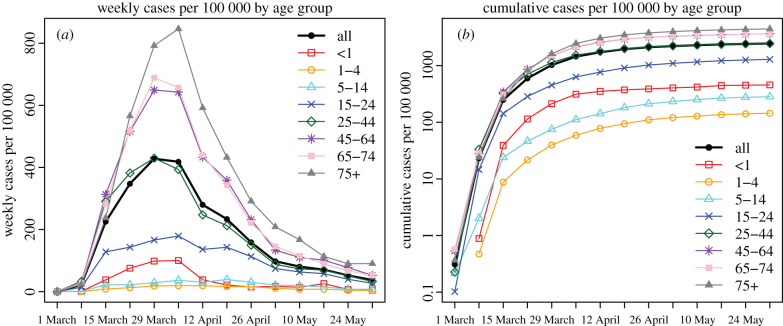
Epidemic dynamics. Reported laboratory-confirmed cases (*a*) and cumulative cases (*b*) per 100 000 population by week of diagnosis for all ages overall and by age group.

Following a diagnosis of the first case in NYC, confirmed COVID-19 cases in the entire population increased nearly exponentially during the first three weeks (figure 1*b*) before slowing down beginning the week of 22 March 2020 when NYC implemented a stay-at-home order. However, case trajectories differed substantially by age group. Foremost, reported cases increased with age: the case trajectory for those aged 25–44 years mirrored the overall epidemic curve, those older than 45 years had higher case rates and those under 25 years had the lowest case rates (figure 1*a* and electronic supplementary material, table S1). Infants (i.e. less than 1 year), however, had higher case rates than 1–4 and 5–14 year olds (figure 1). In addition, the timing of peak case rate was mixed. Case rates in 25–44, 45–64 and 65–74 year olds peaked earliest during the week of 29 March 2020, followed by less than 1, 1–4, 15–24 and 75+ year olds with a 1-week lag; in comparison, the case rate for 5–14 year olds fluctuated with a less clear peak during the weeks of 29 March–26 April 2020.

The epidemic trends based on diagnosed cases, however, were obscured by varying infection detection rates by age and through time. COVID-19 infections are more likely to manifest as symptomatic illness and/or more severe disease in individuals with underlying conditions and in older adults [5,6]. Such differential clinical characteristics by age thus lead to varying healthcare seeking behaviours and infection detection rates by age. In addition, testing policies varied over the course of the spring 2020 COVID-19 pandemic in NYC. During this time, testing capacity was limited at the federal, state and local levels by guidelines for who should be tested (due, for example, to limited availability of test kits, swabbing supplies and reagents), which required prioritizing testing for severely ill patients and those highly vulnerable to severe disease. Testing capacities expanded during the week of 8 March 2020 [13]; however, by late March, material shortages (including testing kits and personal protective equipment) again prompted the city to restrict testing to those severely ill [14]. Using our model-inference system, we estimated that infection detection rates increased in early March, reaching a peak of around 20% for all ages overall during the week of 15 March, and declined afterwards before increasing again in mid-April [11,12].

**Figure 2.**
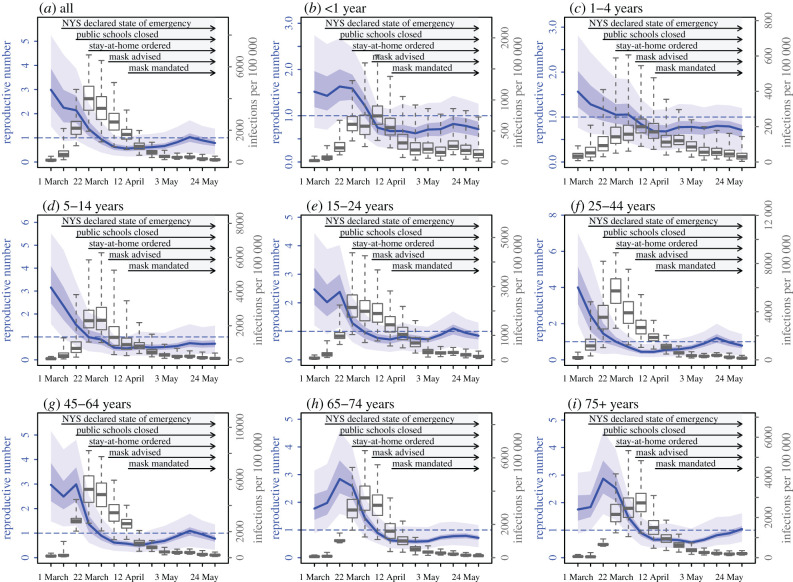
Estimated changes in the effective reproductive number and infection rates. Blue lines show the estimated effective reproductive number (*R_t_*) for each week; surrounding areas show the 50% and 95% CrIs. Superimposed boxes (right *y*-axis) show estimated infection rates by week: median (thick vertical lines), 50% CrIs (box edges) and 95% CrIs (whiskers).

After accounting for infection detection rates to include undiagnosed infections, a different picture of the NYC spring outbreak emerges (figure 2 and electronic supplementary material, figure S3). Estimated infection rates were highest among 25–44 and 45–64 year olds (figure 2*f*,*g*), followed by 65–74, 75+ year olds (figure 2*h*,*i*), then 5–14, 15–24 year olds (figure 2*d*,*e*), and were lowest among less than 1 and 1–4 year olds (figure 2*b*,*c*; electronic supplementary material, table S1). Estimated infection rates in the younger age groups (in particular, 5–14, 15–24 and 25–44 year olds; figure 2*d–f*) peaked during the week of 22 March 2020, followed by the three older age groups (i.e. 45–64, 65–74 and 75+ year olds; figure 2*g*–*i*) about a week later.

Given the large uncertainties in model estimates, we verified our estimates of infection rates using available serology data collected during three phases of the pandemic (i.e. early phase in March [15], mid-phase in April [16] and end of the pandemic wave in June [17]). Overall, our estimated cumulative infection rates were in line with corresponding measures from antibody tests, for all three phases of the pandemic wave (for details, see the appendix of Yang *et al*. [11,12]).

### 2.2. Overall effectiveness of interventions

The reproductive number at time *t* (*R_t_*) measures the average number of persons an infected individual infects and thus reflects underlying epidemic dynamics. The epidemic expands in size if *R_t_* is above unity and subsides otherwise. In addition, when the entire population is susceptible and no interventions are in place, *R_t_*, referred to as the basic reproductive number (*R*_0_), reflects the transmissibility of an infection in that population. Here, we estimated that *R_t_* was 2.99 (median and interquartile range (IQR): 2.32–3.86; electronic supplementary material, table S2) during the first week of the pandemic (i.e. the week of 1 March) in NYC, similar to *R*_0_ estimates reported for other places [18,19]. It decreased to around 2.2 during the next two weeks, when NY State declared a state of emergency and public awareness and voluntary precautionary measures (e.g. avoiding public transit [20]) increased (figure 2*a*). Following the stay-at-home mandate starting the week of 22 March *R_t_* dropped substantially to 1.37 (IQR: 1.08–1.68) during that first week, to 0.93 (IQR: 0.73–1.13) a week later, and to a minimum of 0.56 (IQR: 0.45–0.67) during the week of 12 April (figure 2*a*). These prompt decreases in *R_t_* from mid-March to mid-April indicate that implemented public health messaging and interventions were effective in curtailing COVID-19 transmission.

Similar decreases in *R_t_* occurred among most age groups (figure 2*b*–*i*). Overall, *R_t_* among younger age groups (less than 45 years) decreased one or two weeks earlier than older age groups (45–64, 65–74 and 75+; figure 2*c*–*f* versus figure 2*g*–*i*). Of note, among the four age groups with higher contact rates [21] (i.e. 5–14, 15–24, 25–44 and 45–64 year olds), *R_t_* dropped below 1 the earliest among 5–14 year olds (0.99, IQR: 0.74–1.30; electronic supplementary material, table S2) during the week of 22 March. This is consistent with the earliest public health interventions to this age group: the closure of public schools beginning the week of 15 March [7].

### 2.3. Effectiveness of reducing contact via school closure and voluntary or mandated stay-at-home measures

**Figure 3.**
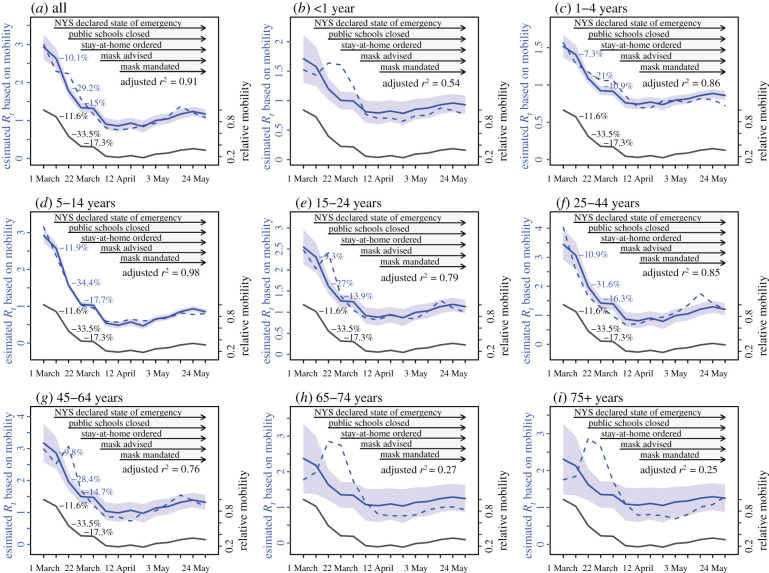
Effectiveness of reducing contact rates. Note the estimated effectiveness combined all measures that reduce contact rates, including school closures and voluntary or mandated stay-at-home measures. Dark grey lines show the observed changes in mobility (right *y*-axis). Blue lines show *R_t_* estimated using a linear regression model with mobility as the sole predictor; surrounding areas show the 95% CrIs of the model estimates. The adjusted *r*^2^ for the regression model is also shown in each plot. For comparison, dashed blue lines show *R_t_* estimates from the model-inference system, without accounting for susceptibility. Percentages attached to the lines show the incremental reductions in either estimated *R_t_* (in blue) or mobility (in grey).

Several public health interventions were implemented around the same time (figure 2), and some interventions may take longer to produce an effect (e.g. due to slower compliance with the measure). It is thus challenging to separate the impact of different interventions. However, a number of measures—including voluntarily working from home during the early weeks of the pandemic, school closures and the stay-at-home mandate—in effect reduce rates of close in-person contact, a key factor for COVID-19 transmission. Thus, here we focus on estimating the impact of interventions whose primary mechanism of action is through a reduction in population contact rates. Given the difficulties measuring this quantity directly, we instead approximated population contact rates using human mobility data, which record real-time population movement based on location changes of individual mobile devices (see Data). Indeed, the reduction in *R_t_* mirrored the reduction in mobility (figure 3). The Pearson correlation (*r*) between *R_t_* and mobility over the 14-week study period was 0.96 for all ages overall and ≥0.9 for 1–4, 5–14, 15–24 and 25–44 year olds (electronic supplementary material, table S3). Thus, we focused on mobility as a proxy for contact rates and used this quantity to estimate the corresponding changes in *R_t_* and segregate the impact of interventions that reduce population contact rates from other concurrent interventions.

Mobility reduced by 11.6% during the second week of the pandemic in NYC (i.e. the week of 8 March), and by a further 33.5% and 17.3% in the following two weeks, respectively (figure 3). Using observed mobility data (i.e. our proxy for population contact rates) to estimate the corresponding changes in *R_t_*, we estimate that, for all ages overall, reductions in population contact rates were associated with *R_t_* reductions of 10.1% (95% CI: 8.3–11.9%) by the second week of the pandemic, and another 29.2% (95% CI: 24.9–33.5%) and 15.0% (95% CI: 14.3–15.8%) in the following two weeks, respectively (figure 3*a*). By the week of 12 April when *R_t_* reached its minimum, the reduction in population contact rates was associated with an *R_t_* reduction of 70.7% (95% CI: 65.0–76.4%). In addition, analysis at the neighbourhood level consistently showed large reductions in *R_t_* that were likely due to reductions in population contact rates (range of median estimates: 66.1–90.1% across the 42 neighbourhoods in NYC; electronic supplementary material, figure S4).

In addition, transmission in four age groups (i.e. 5–14, 15–24, 25–44 and 45–64 year olds) appeared to be most impacted by changing population contact rates (figure 3 and table 1). The reduction in population contact rates was associated with decreases of the age-specific *R_t_* by 83.4% (95% CI: 80.1–86.7%) for 5–14 year olds, 65.4% (95% CI: 57.0–73.8%) for 15–24 year olds, 76.5% (95% CI: 68.5–84.6%) for 25–44 year olds and 68.9% (95% CI: 59.2–78.6%) for 45–64 year olds, by the week of 12 April.

**Table 1.** Estimated effectiveness of reducing contact rate and face covering. Numbers are the estimated mean and 95% CIs. Note the estimated effectiveness of contact rate reduction combined all measures that reduce contact rates, including school closures and voluntary or mandated stay-at-home measures.

age	estimated effectiveness of intervention (%)		
	contact rate reduction	face covering (first month)	face covering (2 months)
all	70.7 (65.0, 76.4)	6.6 (0.8, 12.4)	3.4 (−1.9, 8.6)
<1	53.8 (41.6, 66)	9.3 (−4.2, 22.9)	12.8 (0.2, 25.3)
1–4	51.0 (45.8, 56.2)	0.9 (−5.5, 7.4)	6.7 (0.6, 12.8)
5–14	83.4 (80.1, 86.7)	3.0 (−0.5, 6.6)	1.6 (−1.6, 4.8)
15–24	65.4 (57.0, 73.8)	4.3 (−2.8, 11.4)	4.0 (−2.5, 10.6)
25–44	76.5 (68.5, 84.6)	4.5 (−0.6, 9.7)	−1.0 (−5.6, 3.7)
45–64	68.9 (59.2, 78.6)	8.1 (−0.1, 16.1)	4.4 (−2.9, 11.8)
65–74	55.8 (34.5, 77.2)	20.8 (−0.1, 41.6)	18.3 (−0.2, 36.9)
75+	53.8 (32.3, 75.3)	20.8 (−0.9, 42.5)	16.2 (−3.3, 35.7)

### 2.4. Effectiveness of face covering/masking under real-world conditions

**Figure 4.**
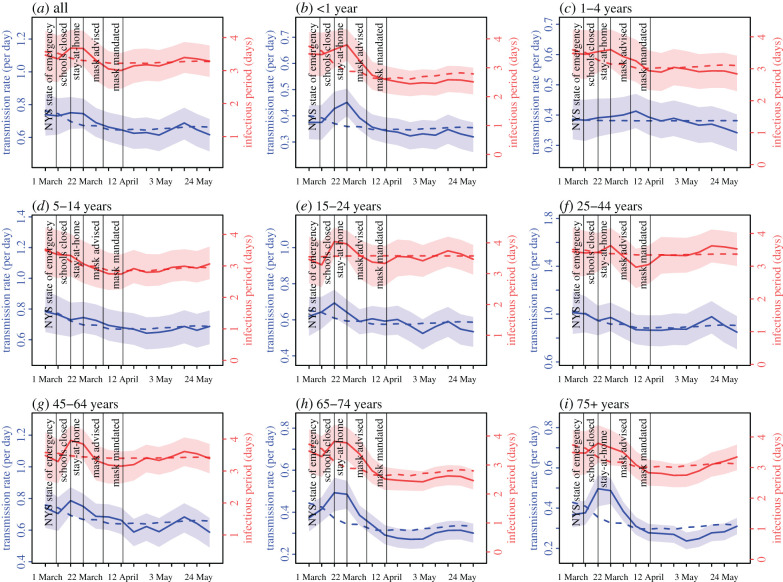
Effectiveness of face covering in reducing the transmission rate and infectious period. Solid lines show the estimated transmission rate (in blue, left *y*-axis) and infectious period (in red, right *y*-axis) using the model-inference system incorporating interventions including face covering. Surrounding areas show the 50% CrIs of model estimates. Dashed lines show corresponding estimates from a linear regression model with mobility as the sole predictor (i.e without accounting for face covering).

Estimated transmission rates (or probability of infection) and the infectious period also closely tracked changes in mobility (electronic supplementary material, table S3; *r* ≥ 0.5 for most age groups). Thus, it appears that reducing mobility not only reduces contact rates but also likely reduces (i) the probability of transmission per contact due to, for example, increased public spacing and (ii) the effective infectious period per infected individual due to, for example, more time spent at home and as a result reduced time for community transmission despite likely unchanged duration of viral shedding. Given this observation, we hypothesize that the relationship between mobility and estimated transmission rates (and effective infectious period) can be used to disentangle the impact of interventions that reduce population contact rates (particularly, the stay-at-home mandate) and face covering/masking—two major public health interventions implemented in NYC during the pandemic—on transmission. We make two predictions if this hypothesis holds. First, predicted transmission rates (infectious period) using mobility data alone would be higher (longer) than those estimated by the model-inference system additionally based on case and mortality data *for weeks when face covering in public was mandated* as it would lead to further reductions in transmission (i.e. temporality and direction of the impact). Second, while the efficacy of masking (i.e. measured under ideal conditions of mask quality and correct use) likely does not vary by individual, the effectiveness of masking (i.e. measured under real-world, often imperfect conditions) and impact of this intervention could vary by subpopulation due, for example, to different usage rates of masks; as such, we expect the predictive errors to be larger for age groups with higher compliance of masking (i.e. magnitude of the impact). Our analyses largely confirmed both predictions. For the first, as shown in figure 4, transmission rates predicted using a linear regression model with the observed mobility as the sole predictor were higher than those estimated by the model-inference system, following the face covering mandate starting the week of 12 April [9]. For the latter, the discrepancies in the two model estimates (i.e. the gaps between the dashed and solid blue lines; figure 4) appeared to increase with age and were largest among the two elderly age groups who have been reported to more frequently use masks [22–24]. However, infants (less than 1 year) appeared to have a larger reduction than other children groups; this could have been due to transmission reduction related to their sources of infection (e.g. their caretakers and healthcare settings where they tended to be exposed). Similar patterns held for the effective infectious period (figure 4).

Given these observations, we further used the discrepancies in the two model estimates to approximate the impact of face covering on reducing COVID-19 transmission. Combining the reduction in the transmission rate and effective infectious period, we estimated that, for all ages combined, face covering contributed to a 6.6% (95% CI: 0.8–12.4%) reduction during the first month it was implemented and a 3.4% (95% CI: −1.9–8.6%) reduction over the entire 8 weeks prior to the city's reopening (table 1). As expected, the estimated impact varied substantially by age group. The effectiveness was 20.8% (95% CI: −0.1–41.6%) for 65–74 year olds and 20.8% (95% CI: −0.9–42.5%) for 75+ year olds during the first month and remained at similar levels afterwards. For 25–44 and 45–64 year olds, two age groups with the highest infection rates (figure 2), the effectiveness was 4.5% (95% CI: −0.6–9.7%) and 8.1% (95% CI: −0.1–16.1%) in the first month, respectively; however, it reduced substantially afterwards, likely due to reversed risk behaviour. Of note, in addition to the likely lower usage rate of face covering in late May–early June, increases in risky behaviours such as large gatherings at the time [25] may have partially obscured the effectiveness of masking.

### 2.5. Retrospective projections of cases and deaths

**Figure 5.**
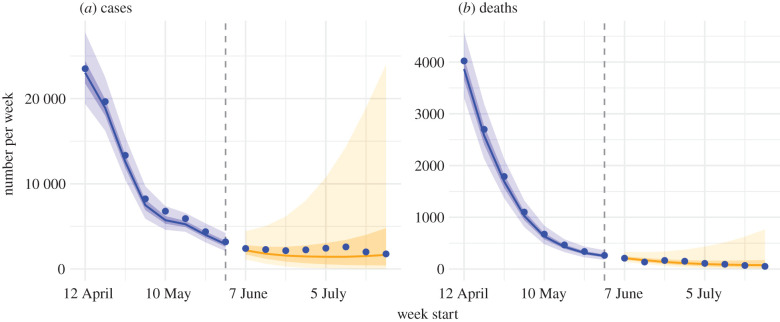
Projections of COVID-19 cases and deaths eight weeks beyond the study period. Blue dots show confirmed cases by week of diagnosis and deaths by week of death, as observed by the surveillance system. Blue lines show model median estimates; surrounding shades show 50% and 90% CrIs. Orange lines show model projected median weekly cases and deaths; surrounding shades show 50% and 90% CIs of the projection.

NYC started phased reopening from the week of 7 June 2020, which allowed industries to gradually reopen per a four-phase plan [26]. For instance, manufacturing industries were allowed to reopen starting the week of 7 June 2020 (dubbed ‘Phase 1'), whereas real estate was allowed to reopen starting the week of 21 June 2020 (dubbed ‘Phase 2'), and personal care services were allowed starting the week of 6 July 2020 (dubbed ‘Phase 3'). As such, population mobility has increased gradually during this time, which could lead to increased transmission of unknown magnitude. Such changes also offer an opportunity to test the accuracy of our estimates—should the estimated effectiveness of reducing contact rates and using face coverings be accurate, these estimates could be used to anticipate changes in transmission in response to the changing mobility and in turn the epidemic dynamics after reopening. We thus used these estimates to generate projections of cases and deaths for the 8 weeks beyond our study period, and compared the projections to available, independent corresponding observations. Overall, our projections underestimated the total number of cases (relative error of median projections: −27% over 8 weeks; figure 5*a*) but were able to accurately estimate the total number of deaths (relative error: −2% over 8 weeks; figure 5*b*). In addition, the examination of age-grouped projections shows that the underestimation of cases was mostly among younger age groups whose case rates had increased in June (1–4, 5–14, 15–24 and 25–44 year olds; electronic supplementary material, figure S5). These recent increases in young cases may have resulted from more young adults returning to work including some in service industries with high contact rates and, relatedly, sending their children to childcare and/or summer camps due to a lack of caretakers at home (information from NYC Department of Health and Mental Hygiene (DOHMH) community investigation; unpublished). In addition, increased risk behaviours of some young individuals (e.g. large parties without physical distancing [25]) may have also contributed to the increased cases among young adults in late June [27]. Consistently, COVID-19 associated mortality, mostly occurring among older adults continued to decrease and was accurately predicted for different age groups (electronic supplementary material, figure S6).

## 3. Discussion

The spring 2020 pandemic wave in NYC, the first epidemic centre in the USA, provides a test case to study COVID-19 epidemiological characteristics and the effectiveness of public health interventions. Through comprehensive modelling, we have reconstructed the transmission dynamics and estimated the effectiveness of two major interventions, social distancing and mandatory face covering in public. Our results show that reducing contact rates (mainly via school closures and voluntary or mandated stay-at-home measures) likely contributed to the largest reduction in transmission in the population overall (approx. 70%) and for most age groups (greater than 50% for all age groups). Widespread use of face covering likely contributed to an additional approximately 7% overall reduction and up to approximately 20% reduction among 65+ year olds during the first month face covering was mandated in public places. Our findings largely consolidate previous model estimates on the impact of lockdown-like measures [4,28,29] and studies on face covering in reducing COVID-19 transmission. These findings provide insights that can inform COVID-19 mitigation efforts in the coming months before the majority of the population can be protected through mass-vaccination, as well as control strategies for other (re)emerging infections in the future.

Lockdown-like measures where confinement at home is encouraged or mandated through school closures, telework policies, closure of non-essential businesses and stay-at-home orders have been a major control measure to curb COVID-19 spread. In effect, such measures reduce population contact rates and thus transmission. Previous modelling studies estimated that lockdowns reduced COVID-19 transmission (measured by *R_t_*) by 58% in Wuhan, China [4], 45% (95% CI: 42–49%) in Italy [29] and 77% (95% CI: 76–78%) in France [28]. Our estimate for NYC overall (approx. 70%) is consistent with these previous estimates. In addition, our estimates show that reducing population contact rates effectively reduced transmission across all age groups (ranging from a 51% reduction among 1–4 year olds to 83% among 5–14 year olds; table 1). Together, these findings underscore the importance of reducing contact rates through, for example, physical distancing in places with continuous community transmission of COVID-19.

The use of surgical masks or cloth face coverings has been another major preventive measure for COVID-19. Studies overall have shown that surgical masks could substantially reduce onward transmission albeit with a large range of efficacy estimates across settings [30]. However, it remains unclear the overall effectiveness of universal face covering requirements at the population level, especially during a pandemic, due to several factors: (i) the overall effectiveness depends on compliance which may vary across subpopulations and time; (ii) improper use of face coverings (e.g. without covering the nose and/or mouth or improper handling [31]) can reduce the effectiveness of face covering; (iii) face coverings are required and mostly worn in public and thus likely have a lower impact in private settings, particularly in reducing household transmission; consequently, the relative impact of face covering depends on the relative contribution of different sources of transmission (e.g. household versus community) at a given time and *vice versa*; and (iv) use of face coverings may lead to complacency and less stringent adherence to social distancing and stay-at-home behaviours. Here we estimated an approximately 7% reduction in overall transmission during the first month of the face covering mandate. However, the estimated effectiveness varied largely across age groups with much higher effectiveness among older adults (approx. 20% for both 65–74 and 75+ year olds versus less than 10% for other age groups). This discrepancy was likely due to the differential compliance and types of face covering used. Observational studies in Wisconsin and surveys nationwide in April/May reported about twofold higher rates of face covering usage among older adults versus younger adults and minors [22–24]. In addition, due to the shortage of surgical masks during March–May [32], older adults at higher risk of severe COVID-19 infection were more likely to use surgical masks whereas younger age groups more frequently used non-medical cloth coverings, which are often less effective [33,34] (e.g. measured ultrafine filtration efficiency is approximately 50% for surgical masks versus approximately 10–25% for T-shirt and approximately 25–35% for cotton covers [34]).

When lockdown-like measures are lifted, residents will spend more time outside their homes than during the lockdown. Adjusting for the time spent outside of homes (approx. 8.3 h in April 2020 versus approx. 11.5 h in June–July 2020 and approx. 13.5 h pre-pandemic; NYC data [35]), universal face covering would have reduced overall transmission by approximately 9–11% (i.e. 6.6% multiplied by a factor of 1.4–1.6) during reopening, given the same rates of face covering as in April. However, if the same effectiveness among older adults were achieved among other age groups, universal face covering could reduce overall transmission by up to approximately 28–32% (i.e. 20% multiplied by a factor of 1.4–1.6). The implication of this latter estimate is twofold. On the one hand, it suggests that for places with the high level of transmission, implementing face covering *alone* is likely insufficient to lower the effective reproductive number *R_t_* to less than 1 in order to control the epidemic (for instance, for a city with an *R*_0_ = 3, the resulting *R_t_* would be 3 × (1–30%) = 2.1). This finding is consistent with the observed resurgence of COVID-19 cases in NYC during autumn/winter 2020 despite the concurrent high usage rate of face coverings (approx. 90% of survey respondents in NYC reported always or frequently wearing masks in public in July 2020 [36], and this number was likely higher during autumn/winter 2020). On the other hand, our findings also suggest that improving effective usage rates of face coverings, especially among younger age groups, could significantly mitigate the risk of a resurgence of COVID-19 infections during re-opening (i.e. approximately 30% reduction without compromising economic growth). It is thus crucial for future research to understand reasons for the use/non-use and selection of face coverings by age group to inform strategies to increase consistent and correct mask use in settings where social distancing is not possible.

It is important to note, however, that not all individuals have the same opportunities to physically distance and/or adopt face coverings during a pandemic, despite government mandates. For instance, over one million frontline workers in NYC (e.g. healthcare workers, transportation workers, janitors, and grocery clerks, which comprise 25% of the city's workforce) had to continue their essential work during the pandemic [37]. In addition, data from the US Bureau of Labor Statistics suggest Black and Latino communities have less opportunities to work from home [38]. Consequently, NYC neighbourhoods with more frontline workers and/or Black and Latino residents tended to have lower reductions in population mobility during the pandemic [35]. In NYC, these communities also experience a number of social conditions that are thought to exacerbate COVID-19, including overcrowded multigenerational households, poverty and high prevalence of chronic diseases. These communities, known to also carry a higher burden of underlying health conditions, suffered greater impacts from COVID-19 and have expressed fear and experiences of racialized bias when wearing a face covering [39,40]. Further research is warranted to investigate such health disparities. In addition, future policies should take into account structural inequities in labour trends, overcrowded housing, and underlying conditions and adopt additional preventive measures to protect those vulnerable communities.

We also note there remain large uncertainties in our estimates due to several limitations. First, we used population mobility as a proxy for contact rates rather than more direct measures. Similar approximation and uncertainty applied to our estimates of the effectiveness of face covering. Future studies are thus warranted for further assessment. For instance, large population-scale surveys documenting changes of the intensity and pattern of contact during the pandemic could provide more accurate measures of contact rates among different age groups and over time. Second, while we restricted our analysis on the effectiveness of face covering to a period when masks were mandated, there remain other residual confounding effects. For instance, increased awareness of COVID-19 and health risk among key age groups such as the elderly may have contributed to further reductions of transmission through other precautions in addition to face covering; this may have led to an overestimation of the effectiveness of face covering for those age groups. Third, here we focused on estimating the effectiveness of interventions in the general population without segregating key settings with intense transmission (e.g. long-term care facilities). Future studies should assess the impact of interventions targeting such high-risk settings. Lastly, our estimates here were largely based on the first wave of the pandemic and may not fully capture subsequent changes in awareness and perception of COVID-19 and related behavioural adjustment during later waves. However, we have also used a similar methodology to estimate the effectiveness of reducing contact rates and face covering under different city reopening schedules and generated long-term projections for NYC; the projections generated during June 2020 for the period of June 2020–May 2021 have been consistent with observations up to the end of 2020 (i.e. at the time of this writing; see the projected resurgence and second wave in Yang *et al*. [41] and comparison with available data in electronic supplementary material, figure S7). These results thus support the robustness of our estimates here.

Our study also has several strengths. In particular, our estimates were based on comprehensive model-inference incorporating multiple data streams and further evaluated using model projections. Our results thus provide an assessment of two major public health interventions (reducing contact rates and face covering) at the population level where the overall effectiveness depends on multiple factors in addition to the efficacy of a given intervention. Altogether, our estimates support the need for multiple interventions (including reducing contact rates by, for example, restricting occupancy, universal face covering and, albeit not studied here, testing, contact tracing, isolation and timely treatment of cases) in order to effectively mitigate the spread of COVID-19 as it continues to pose threats to public health.

## 4. Methods

### 4.1. Data

COVID-19 cases included all laboratory-confirmed cases by week of diagnosis reported to the NYC DOHMH. Mortality data by week of death combined confirmed and probable COVID-19-associated deaths. Confirmed COVID-19-associated deaths were defined as those occurring in persons with laboratory-confirmed SARS-CoV-2 infection, and probable COVID-19 deaths were defined as those with COVID-19, SARS-CoV-2 or a similar term listed on the death certificate as an immediate, underlying or contributing cause of death, but did not have laboratory confirmation of COVID-19 [42]. For this study, both weekly case and mortality data were aggregated by age group (less than 1, 1–4, 5–14, 15–24, 25–44, 45–64, 65–74 and 75+ years) for each of the 42 United Hospital Fund (UHF) neighbourhoods [43], according to the patient's residential address. All data were retrieved on 4 September 2020. For a summary of the spatial variations across the 42 neighbourhoods, see table S3 in the appendix of Yang *et al*. [12].

The mobility data, used to model changes in population contact rates due to public health interventions implemented during the pandemic (e.g. social distancing), came from SafeGraph [35,44] and contained counts of visitors to locations in each zip code from the same zip code and others, separately, based on mobile device locations. The released data were anonymized and aggregated at weekly intervals. We spatially aggregated these data to the UHF neighbourhood level, for both intra- and inter-UHF neighbourhood mobility. In addition, SafeGraph also provided an aggregate measure of the length of time spent outside of the home during each week.

This study was classified as public health surveillance and exempt from ethical review and informed consent by the Institutional Review Boards of both Columbia University and NYC DOHMH.

### 4.2. Network transmission model

The epidemic model used in this study was described in Yang *et al*. [11,12]. Briefly, the model simulated intra- and inter neighbourhood transmission of COVID-19 using a susceptible–exposed–infectious–removed (SEIR) network model:

4.1 $$\left\{ \begin{array}{rl} \frac{dS_{i}}{dt}= & -S_{i}\overset{\;j=42}{\underset{\;j=1}{\sum }}b_{\;j}\beta_{\mathrm{city}}c_{ij}I_{\;j}/N_{\;j},\\ \frac{dE_{i}}{dt}= & S_{i}\overset{\;j=42}{\underset{\;j=1}{\sum }}b_{\;j}\beta_{\mathrm{city}}c_{ij}I_{\;j}/N_{\;j}-\frac{E_{i}}{Z},\\ \frac{dI_{i}}{dt}= & \frac{E_{i}}{Z}-\frac{I_{i}}{D}\\ \mathrm{and}\;\frac{dR_{i}}{dt}= & \frac{I_{i}}{D}, \end{array}\right.$$

where *S_i_*, *E_i_*, *I_i_*, *R_i_* and *N_i_* are the numbers of susceptible, exposed (but not yet infectious), infectious and removed (either recovered or deceased) individuals and the total population, respectively, from a given age group in the neighbourhood *i*. Note that due to model complexity and a lack of information for parametrizing interactions among age groups, we modelled each age group separately (i.e. combining all sources of transmission to each age group; see further detail on parameter estimation below); as such, equation (4.1) describes the spatial transmission across neighbourhoods without interactions among age groups. *β*_city_ is the citywide transmission rate, which incorporated seasonal variation as observed for OC43, a beta-coronavirus in humans from the same genus as SARS-CoV-2 [12]. To allow differential transmission in each neighbourhood, we included a multiplicative factor, *b_i_*, to scale neighbourhood local transmission rates. *Z* and *D* are the latency and infectious periods, respectively (electronic supplementary material, table S4).

The contact rates (*c_ij_*) in each neighbourhood over time and connectivity among neighbourhoods were computed based on mobility data. The model also accounted for delays from infection to diagnosis using two parameters (gamma distribution with mean *T_d_* and standard deviation *T_sd_* estimated along with other parameters) and death (based on the observed time from diagnosis to death) as well as infection detection rate using a parameter *r* (estimated along with other parameters). For further detail, refer to Yang *et al*. [11,12].

### 4.3. Parameter estimation

To estimate model parameters (e.g. *b_i_*, *β*_city_, *Z*, *D*, *r* and *infection fatality risk*, for *i* = 1, … ,42) and state variables (e.g. number of susceptible and infectious individuals in each neighbourhood) for each week, we ran the network model stochastically with a daily time step in conjunction with the ensemble adjustment Kalman filter [45] and fit to weekly case and mortality data from the week starting 1 March to the week ending 6 June 2020. The posterior distribution of each model parameter/variable was updated for that week at the same time [45]. This parameter estimation process was done separately for each of the eight age groups (i.e. less than 1, 1–4, 5–14, 15–24, 25–44, 45–64, 65–74 and 75+ years). To include transmission from other age groups, we used measured intra- and inter-group contacts from the POLYMOD study [21] to compute the total number of contacts made with each age group and adjusted the *prior* range of the transmission rate (*β*_city_) for each age group accordingly. The posterior estimate was computed based on cases and mortality data for each group, which included all sources of infection. Thus, the estimated transmission rate for each age group nevertheless included all sources of transmission. To account for stochasticity in model initiation, we ran the parameter estimation process independently 10 times. Results for each age group were combined from these 10 runs (each with 500 model realizations). We computed age-specific *R_t_*, the effective reproductive number during week *t,* from the posterior estimates of the transmission rate (*β*_city_ and *b_i_*), infectious period (*D*), contact matrix (*c_ij_*), susceptibility and population size in the neighbourhood using the next generation method [46]. We computed *R_t_*, *β*_city_ and *D* estimates for all ages overall as a weighted average of the age-specific estimates with weights equal to the population fraction in each age group.

### 4.4. Estimating the effectiveness of reducing contact rates

The *R_t_* estimates from the model-inference system capture changes in transmission due to various interventions, i.e. the overall effectiveness of all implemented interventions. To separately estimate the effectiveness of interventions that reduce contact rates, we used human mobility as a measure of population contact rate to estimate the changes in *R_t_* in response to changing population contact rates. Specifically, we regressed the *R_t_* estimates from the full model-inference system on the mobility data:

4.2 $$R_{t}=a_{0}+a_{1}M_{\mathrm{ave},t},$$

where *M_*ave*,*t*_* is the mean of all intra-neighbourhood mobility at week *t*. We then computed the effectiveness of reducing contact rate based on $\hat{R_{t}}$, the *R_t_* estimate from this regression model solely based on the observed mobility. That is, the reduction in *R_t_* by week *t*, likely due to reducing contact rate, was computed as

4.3 $$\frac{\hat{R_{t}}-\hat{R_{0}}}{\hat{R_{0}}}.$$

To test the robustness of our method, we performed the same analysis for individual UHF neighbourhoods (*n* = 42).

### 4.5. Estimating the effectiveness of face covering/masking

In addition to changes in population contact rates, face covering/masking was another major control measure implemented beginning the week of 12 April 2020 when NYC mandated residents wear face coverings in public places. To estimate the effectiveness of face covering, we first estimated the changes in transmission rate and effective infectious period, two model parameters determining *R_t_*, due to changes in mobility (as opposed to masking) using regression models similar to equation (4.2). Specifically, we regressed the estimated citywide transmission rate (or effective infectious period) from the full model-inference system on average mobility:

4.4 $$Y_{t}=a_{0}+a_{1}M_{\mathrm{ave},t},$$

where *Y_t_* is *β*_city_ or *D.* We then computed the relative reduction in transmission rate (or effective infectious period) due to face covering as $\eta_{Y}=E((Y_{t}-\hat{Y_{t}})/Y_{t})$, where *E*(·) gives the mean over the relevant timeframe (here we estimated two timeframes, i.e. one month following the mandate and over eight weeks up to 6 June 2020). Combining both reductions, we computed the effectiveness of face covering as *η* = 1 − (1 − *η_β_*)(1 − *η_D_*). Of note, while mechanistically face coverings act primarily by reducing the probability of transmission (i.e. transmission rate), here we included both the potential impact on the transmission rate (*η_β_*) and effective infectious period (*η_D_*), mainly because the multiplicative relationship of the two variables with *R_t_* makes it challenging to separate the two effects. Nevertheless, reductions in the infectious period via face covering are possible. A recent study on animals showed that masking could reduce the severity of infection [47]; if persons with milder infection experience shorter duration of viral shedding (there is some evidence for this, e.g. from He *et al*. [48]), milder symptoms in individuals infected while wearing face covering could lead to shorter infectious period of these individuals.

### 4.6. Projections of cases and deaths

To evaluate the accuracy of model estimates, in particular, the effectiveness of transmission reduction by reducing contact rates and use of face covering, we tested if these estimates along with the model could generate accurate predictions of cases and deaths for eight weeks beyond the study period (i.e. from the week of 7 June 2020 to the week of 26 July 2020). We first projected the citywide transmission rate and infectious period based on observed mobility using equation (4.4); these estimates thus accounted for changes due to changes in contact rates. To incorporate the reduction in transmission by face covering, we further reduced the projected city transmission rate by a factor of 1 − *η_β_p*_out_ and the infectious period by a factor of 1 − *η_D_p*_out_, where *p*_out_ is a factor to adjust for time spent outside of the home during each week. To reflect longer term usage rates of face covering, we used *η_β_* and *η_D_* estimated during the entire eight weeks face covering was required (i.e. from the week of 12 April 2020 to the week of 31 May 2020). Finally, we used estimates of population susceptibility and infection rates at the end of the week of 31 May 2020 to model initial conditions and integrated the SEIR network model forward stochastically for eight weeks using the projected transmission rate and infectious period.

## Data accessibility

COVID-19 case and mortality data for New York City are publicly available at https://github.com/nychealth/coronavirus-data. Note that age and spatial breakdowns of the data may be different from those used in the study.
